# Supplementary material for: Mitochondrial membrane hyperpolarization modulates nuclear DNA methylation and gene expression through phospholipid remodeling
Source: Nat Commun. 2025 Apr 29;16:4029. doi: 10.1038/s41467-025-59427-5 (PMC12041266; doi:10.1038/s41467-025-59427-5)

Supplementary Figure 1. Mitochondrial hyperpolarization via IF1 knockout does not elicit compensation in mtCU or engages AMPK. A) Representative immunoblots of whole cell lysates (n=3; independent clones) from HeLa and HEK293T cells deleted for IF1 using two different guide RNAs. Lane: 1, HeLa WT; 2-4, HeLa IF1-KO (sgRNA1); 5-7, HeLa IF1-KO (sgRNA2); 8, 293T WT; 9, 293T IF1-KO. Upper panel: blots using IF1 antibody, lower panel shows loading control, β-actin B) ΔΨm was measured in intact cells using TMRE; data were normalized to mitochondrial content using MitoTracker® Green (MTG). On *y*-axis, TMRE/MTG ratio, black bar, WT cells, and red bar, IF1-KO cells. Panel on left show data on HeLa cells (n = 7; error bars represent ±SEM); middle and right panels show uncoupling controls. Statistical difference was determined using 2-sided unpaired Student's t test. C) Immunoblots of whole cell lysates from HEK293T cells (n=3 independent biological replicates) and relative quantification of mtCU subunits. Lanes: 1-3, WT cell lysates; 4-6, IF1-KO cell lysates; 7, WT MICU-1 cell lysate (control); 8) MICU1-KO cell lysate (antibody control). Statistical difference was determined using 2-sided unpaired Student's t test. D) Immunoblots of whole cell lysates from HEK293T cells and relative quantification of total AMPK and phosphorylated AMPK (Thr172). Lanes: 1-2, WT cell lysates; 3-4, IF1-KO cell lysates; n=2 independent biological replicates). E) Immunoblots of blue native (BN)-PAGE using digitonin-permeabilized WT (lanes 1-2 in each blot) and IF1-KO (lanes 3-4 in each blot). Predicted size of ATP synthase is pointed by the arrow. Left panel: WT and IF1-KO mitochondria blotted for ATP5O, middle and right panels, respectively, samples were blotted with antibodies for ATP8 or ATPβ. Experiments were repeated 3 times with n=2 independent biological replicates.

Supplementary Figure 2. Characterization of mitochondrial structure and function in IF1-KO cells. A) Immunoblots of whole cell lysates from HEK293T cells and relative quantification of IF1 levels. Lanes: 1-3, WT; 4-6, IF1-KO; 7-9, IF1-OE (for overexpression). Upper panel, IF1 antibody. Lower panel, β-actin antibody. Graph on the right shows the quantification of the blots; n=3 independent biological replicates. B) Oxygen consumption rate (OCR) and extracellular acidification rate (ECAR) of WT, IF1-KO, and IF1-OE cells with different oxidizable carbon sources. Left graphs: cells grown in glucose 4.5 g/L (25 mM); right graph: cells grown in galactose 0.9 g/L (5 mM); upper graphs, OCR on the *y*-axis in pmol O_2/min/µg protein; lower graphs, ECAR: on the *y*-axis in mpH/min/µg protein. On the *x*-axis, time in minutes (min). Arrows indicate injection of mitochondrial inhibitors: oligomycin A 1 µM (O), FCCP 1 µM (F), and rotenone and antimycin A 1 µM (R/A). Data are presented as mean values ±SEM of n=4 biological replicates. C) Graphs depict data derived from OCR measurements: Basal mitochondrial respiration, ATP-linked respiration, maximum respiration, spare respiratory capacity, H^+ leak, and non-mitochondrial respiration. *y*-axis, pmol O_2/min/µg protein. Data are presented as mean values ±SEM of n=4 biological replicates Statistical difference by 2-sided one-way ANOVA with Tukey's post-test. D) Representative electron micrograph of WT, IF1-KO and IF1-OE cells. Upper images at 18,500X magnification, scale 2 µm; bottom images at 49,000X magnification, scale 500 nm. E) Measurements of mitochondrial perimeter from EM images, error bars represent the mean ± SD (n = 63). Statistical difference by 2-sided one-way ANOVA with Tukey's post-test. F) WT, KO and OE were exposed to FCCP to induce mitophagy, immunoblots for cleaved LC3A (I and II) are shown in the upper panels; loading control (GAPDH) is shown in the lower panel;

data are representative of n=3 independent biological replicates. G) Bar graph of ΔΨm in cells grown in phosphate-depleted medium for 72h. *Y*-axis depicts the ratio of TMRE to MitoTracker Deep Red (MTDR), which is insensitive to ΔΨm. Data are presented as mean values ±SEM of n=3 biological.

Supplementary Figure 3. Mitochondrial membrane hyperpolarization is associated with genome-wide DNA hypermethylation. A) Schematic depiction of genomic regions commonly assigned for probes from methylation arrays. TSS (transcription start site) +200 and +1500 were chosen to depict differentially methylated loci (DML) at the promoter regions. B) Pie charts depicting degree of methylation difference (based on average beta-β -values) in all regions of the genome analyzed. Pie chart on the left represents the WT cells; right chart represents data from the IF1-KO counterparts. Table below charts provides specifics on mean methylation in each genomic region as a function of genotype; difference between the means and statistical parameters are also shown. Mann-Whitney U test was applied. C) Scatter plots showing genes that are differentially methylated based on their TSS status (data from Fig. 3A). In blue: genes that are hypomethylated and upregulated, in green: genes hypermethylated and upregulated, in yellow: hypomethylated and downregulated genes and, in red, those that are hypermethylated and downregulated.

Supplementary Figure 4. Decreasing mitochondrial membrane hyperpolarization reverses the DNA hypermethylation phenotype. A) Bar graph of ΔΨm measurements in WT, KO and KO cells treated with dinitrophenol (DNP), a mild uncoupler, for 3 days; ΔΨm was measured by TMRE staining. *Y*-axis depicts the TMRE/MTG ratio. (n = 6 independent biological replicates/genotype); error bars represent ±SEM. Statistical

difference by 2-sided one-way ANOVA with Tukey's post-test. B) Representative immunoblots of whole cell lysates from IF1-KO cells overexpressing UCP4 (*SLC25A27*). Lanes: 1, WT cell lysate; 2, IF1-KO cell lysate; 3-9, IF1-KO UCP4 clones cell lysates. Upper panel, UCP4 antibody. Lower panel, β-actin antibody. Results were reproduced independently twice. C) Bar graph of $\Delta\Psi m$ as measured by TMRE staining. *Y*-axis depicts the TMRE/MTG ratio. (n = 3); error bars represent ±SEM. Statistical difference was determined using 2-sided unpaired Student's t test. D) Table compiling the number of differentially methylated loci (DML) that reversed in IF1-OE or IF1-KO UCP4 cells fully or partially. The number of loci that had no DNA methylation change is also shown. In each genotype, full reversal represents that methylation status of the locus in the OE or UCP4 cell was different than the KO but not than the WT isogenic counterparts. Partial reversal is defined based on locus methylation that was statistically different than IF1-KO and in the opposite directionality of IF1-KO vs. WT comparison; it is also statistically different than WT. E) Heatmap depicting promoter-specific DNA methylation status in the KO vs OE (left panel) or KO vs UCP4 (right panel). Each line represents the same locus in the different genotypes. Shades closer to yellow indicate hypermethylation while those closer to purple hypomethylation. These data derive from the list of DML at TSS shown in Supplementary Data 3.

Supplementary Figure 5. Phospholipids correlate with nuclear DNA, but not histones, hypermethylation. A) Immunoblots on whole lysates of n=3 biological replicates of WT, IF1-KO and IF1-OE cells. DNA methyltransferases (DNMTs), Ten Eleven Translocation (TET) and Thymine DNA Glycosylase (TDG). GAPDH and actin were used as loading control. Bar graphs show quantifications with error bars ±SEM;

significance was determined with 2-sided one-way ANOVA and Tukey's post-test. B) Differentially enriched metabolites (FDR > 0.05) from untargeted metabolomics. Shades of red and blue represent, respectively, increased or decreased levels (log2FC). Spearman's p, Pearson's r and coefficient of determination were used as statistical metrics with $p<0.05$. C) Phospholipids identified through untargeted metabolomics. On the *y*-axis, -log10 FDR. On the *x*-axis, log2FC. Blue: phosphatidylcholine (PC); red: phosphatidylethanolamine (PE). D) Heatmap of differentially enriched phospholipids (FDR > 0.05) grouped by head groups; shades of red are increased, and shades of blue are decreased levels. E) PC/PE ratio of pooled PC/PE pairs (16:0/18:2, 16:0/22:6, 18:0/22:6, and 18:2/18:2) from metabolomics; On the y-axis, FC of metabolites relative to WT; n = 5, significance by 2-sided One-way ANOVA with Tukey's post-test; error bars represent ±SD. F) Representative HPTLC plate: increased series of phospholipid (PL) standards (lanes 1-4); lanes 5-10: isolated mitochondria loaded at increasing amounts. G) Total (left graph) or mitochondrial (right graph) PS content from HPTLC; n=6/genotype; One-way ANOVA with Tukey's post-test. H) Immunoblots of n=3 independent purified histone preparations. I) PEMT normalized counts in different cell lines, HEK293 circled in red, as per the Proteinatlas.org. J) Total methionine was set as 100% for each sample and isotopologues +0 and +3 were calculated as percentage (%) from the total. On the *y*-axis, relative abundance, on the *x*-axis, isotopologue type. (n = 3; statistical difference by 2-sided unpaired Student's t-test). K) Immunoblots probing flag tagged-PEMT expressed in WT (lane 1) and IF1-KO (lane 2); lane 3: IF1-KO alone. L) DML found between IF1-KO and IF1-KO-PEMT; each line represents the same TSS. Shades of yellow:

hypermethylation, shades of purple: hypomethylation. M) Genes associated with PC metabolism through the Kennedy pathway based on RNA-seq (Supplementary Data 1).

Supplementary Figure 6. Environmental chemicals hronically hyperpolarize mitochondria without toxicity. A) Nine chemicals that were shown to acutely change JC10 fluorescence as per Tox21 (tox21.gov) were tested for acute (15 min) or chronic (10 days) modulation of ΔΨm as judged by TMRE/MTG fluorescence. For acute treatments, cells were exposed to the chemicals in the presence of TMRE/MTG; for chronic experiments, WT HEK293 cells were exposed for 10 days to the test articles and loaded with TMRE/MTG at the time of the measurements. Monensin was toxic after 6 days; all chemicals indicated as NA were tested acutely but were not tested under chronic exposure conditions. B) Crystal violet stain was used to determine cytotoxicity of chronic exposures to DMSO (vehicle) or the drugs telmisartan or annatto. Images are derived from 3 independent experiments; the quantification of cell survival is depicted in the bar graph below the images; error bars represent ±SD.

Supplementary Figure 7. Ovarian cancer cell lines with *ATP5IF1* (IF1) copy number alteration. Publicly available data on Ovarian Cancer Cell Lines was searched using cBioPortal.org. A) Schematic representation of deep deletion of the IF1 gene in OVCAR3 cells. No copy number variation, mutation or structural variants in the IF1 locus was identified in the ovarian cancer cell line of the same sub-type and grade Caov3. B) IF1 mRNA expression levels from RNA-seq in both cell lines as per data deposited on cBioPortal. C) IF1 protein abundance from proteomics of different ovarian cancer cell lines categorized in two groups (based on copy number): i) deep deletion and ii) no

alteration (diploid). OVCAR3, larger brown circle in deep deletion group; Caov3, larger blue circle in no alteration (diploid) group.

Supplementary Figure 8. Inactivation of the pentose phosphate pathway in IF1-KO cells leads to complete cell cycle arrest with features of cellular senescence. Glucose-6-phophate dehydrogenase (G6PD), the rate limiting enzyme of the PPP, was deleted by CRISPR/Cas9 in the IF1-KO cells. Representative phase contrast images of cell number and morphology in WT, IF1-KO WT for G6PD and IF1 double KO; images were taken 7 days after single clone selection. Western blots probing for G6PD, p21 and IF1; actin was using as loading control. Data re representative of n=3 independent biological replicates that were assessed at independent times.

Supplementary Data 1. Differentially expressed genes. RNA-seq was performed in the cells using n=4 independent biological replicates. Differential expression analysis was performed in the R statistical programming environment v4.1.2 using DeSeq2 v1.34.0 (https://www.r-project.org/)[64]. Genes with an adjusted *p*-value < 0.05 were classified as differentially expressed. Benjamini-Hochberg procedure was applied for multiple testing correction.

Supplementary Data 2. Differentially methylated loci and differentially methylated expressed genes between IF1-KO cells and the WT counterparts. Whole genome DNA methylation levels was probed using the Illumina 850K array. A total of n=4 independent biological samples per genotype were submitted for the arrays. Hypermethylated or hypomethylated probes were defined by a change in mean beta (β) value >10 or <10, respectively, and an adjusted *p*-value <0.05. The Benjamini-Hochberg procedure was applied to adjust p-values for multiple testing correction.

Supplementary Data 3. Differentially methylated loci in IF1-KO cells ectopically expressing IF1, UCP4 or PEMT relative to the IF1-KO isogenic counterpart. Whole genome DNA methylation levels was probed using the Illumina 850K array. For each genotype, n=4 independent biological samples were submitted for the arrays. Hypermethylated or hypomethylated probes were defined by a change in mean β value >10 or <10, respectively, and an adjusted *p*-value <0.05. The Benjamini-Hochberg procedure was applied to adjust p-values for multiple testing correction.

Supplementary Data 4. Analysis of TEs and H3K9me3 as proxy for heterochromatin changes caused by IF1 loss. Comparison of transposable element (TE) expression between WT and IF1-KO cells was performed as described in the methods. H3K9me3 peaks and their overlap with the promoters of DEGs was done using Hg38 H3K9me3 HEK293 data from the ENCODE project; coordinates of DEG promoters was defined as ±1.5 Kb of the annotated transcription start site (TSS). More details in the methods section.

Supplementary Figure 1

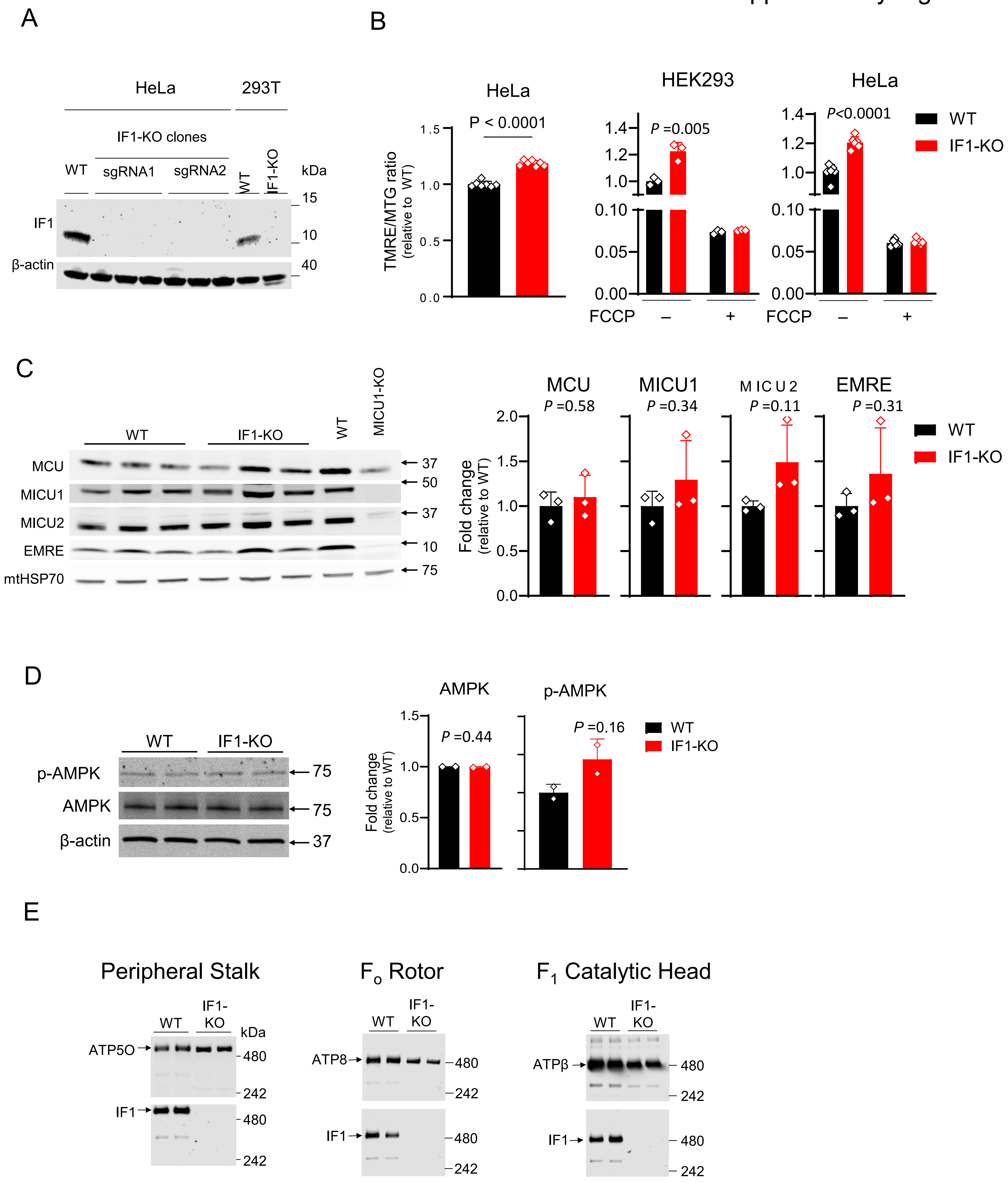

Supplementary Figure 2

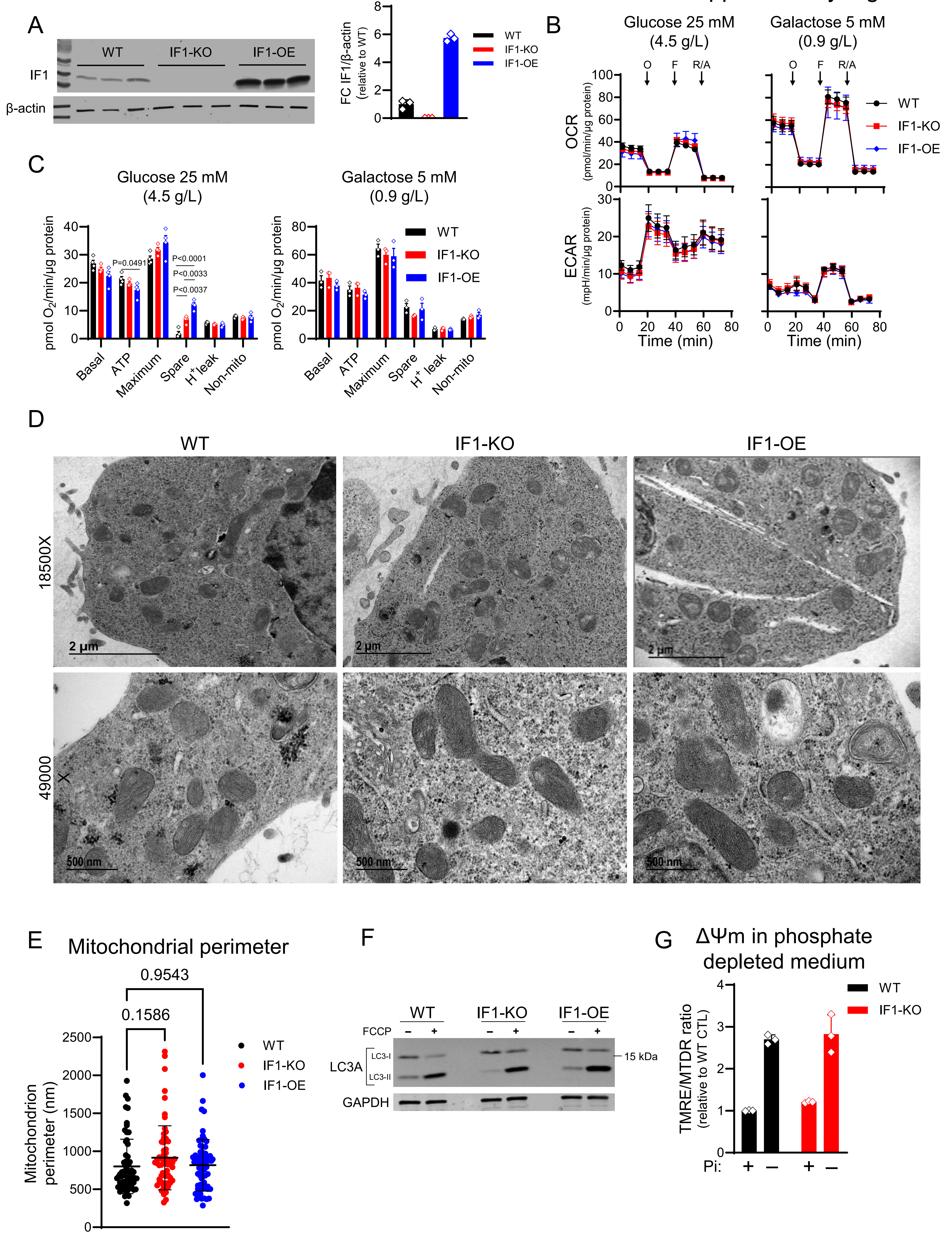

Supplementary Figure 3

A

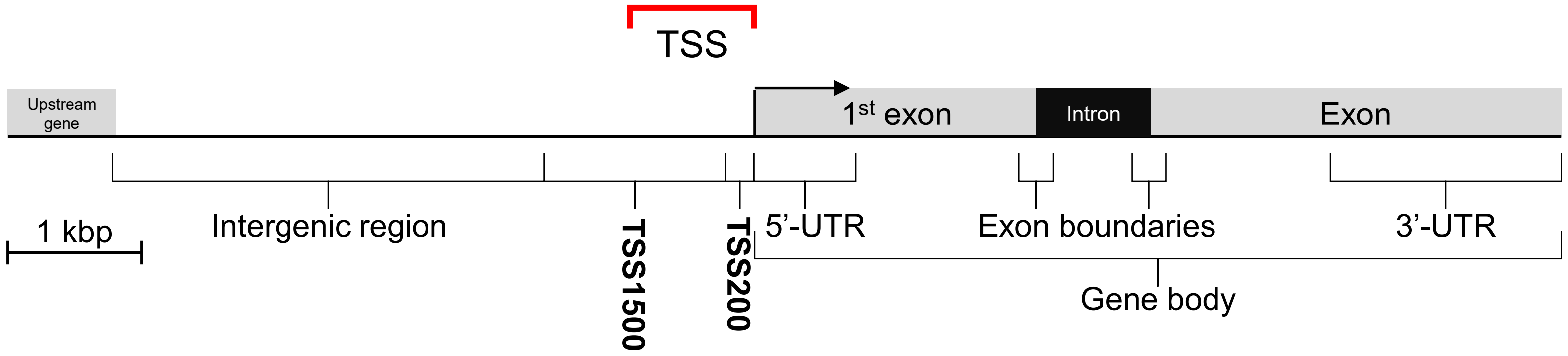

B

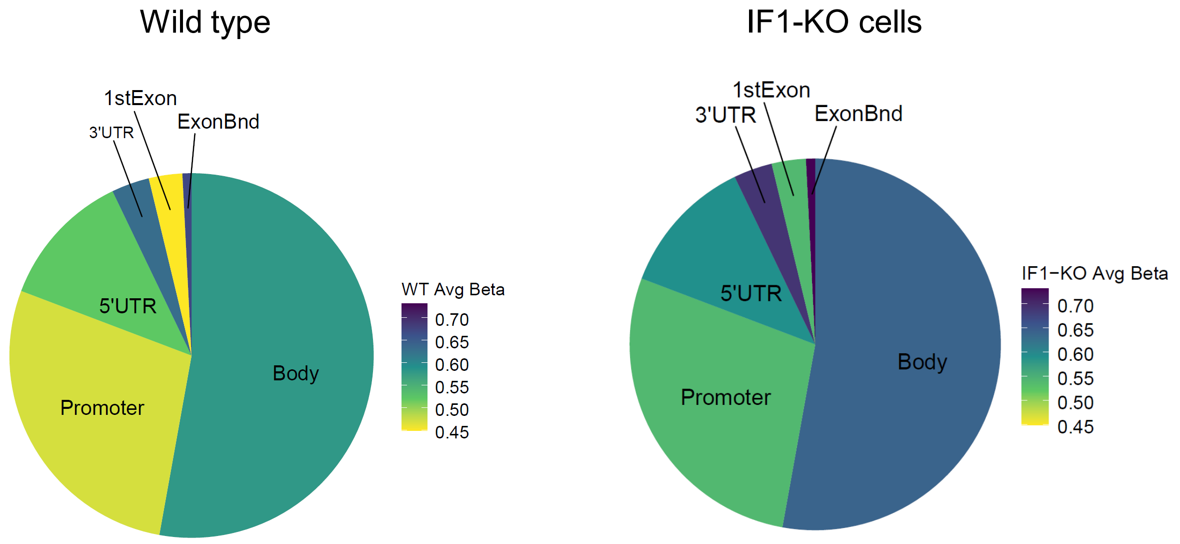

Means	Feature	ko.mean	wt.mean	diff.mean	p.value	effect.size	alternative
	1stExon	0.5351703	0.453751	0.0814192	4.20E-29	0.2760179	two.sided
	3'UTR	0.6948496	0.6256567	0.0691929	7.60E-41	0.2665405	two.sided
	5'UTR	0.5862812	0.518112	0.0681692	2.55E-83	0.2355359	two.sided
	Body	0.6393725	0.5754339	0.0639386	0	0.2283931	two.sided
	ExonBnd	0.7254582	0.6674032	0.058055	4.43E-08	0.2328262	two.sided
	IGR	0.5862575	0.5325107	0.0537468	1.74E-216	0.1880556	two.sided
	TSS1500	0.5470964	0.4772081	0.0698883	1.90E-143	0.237614	two.sided
	TSS200	0.5227778	0.4383732	0.0844046	2.00E-74	0.2866383	two.sided
	All DMPs	0.5982983	0.5354602	0.0628381	< 2.2e-16	0.2180837	one.sided (KO>WT)

C

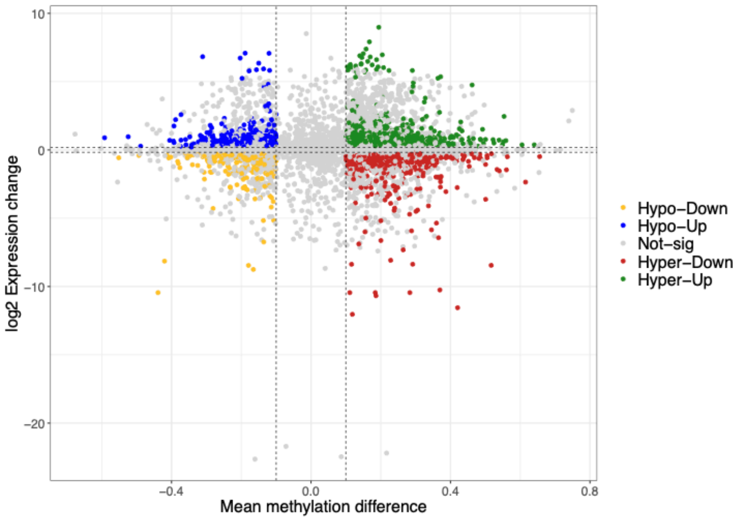

Supplementary Figure 4

A

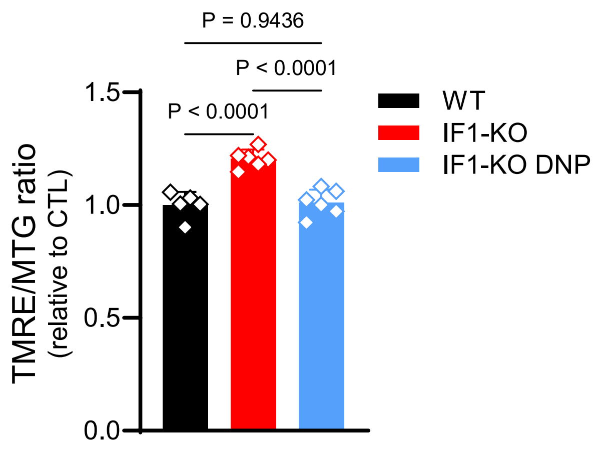

B

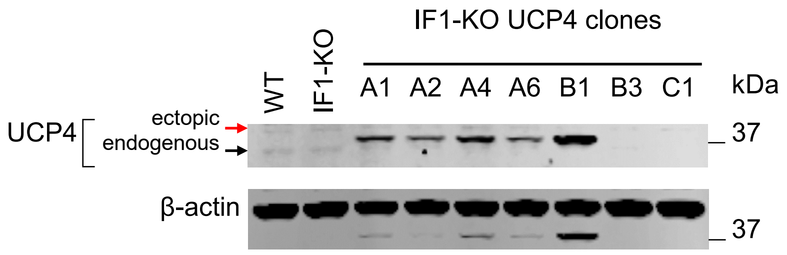

C

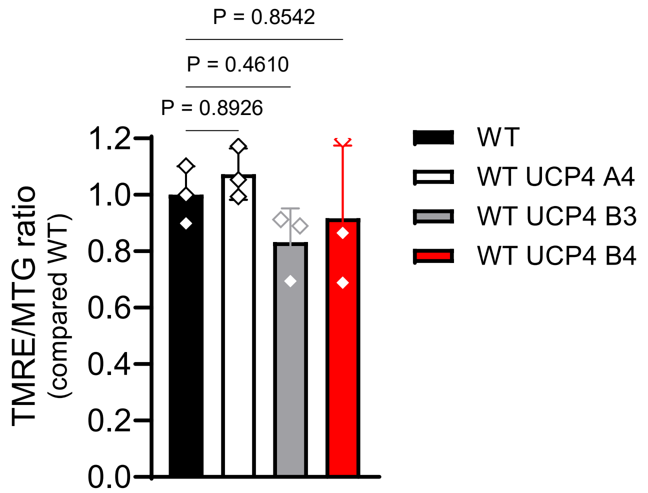

D

DNA methylation changes in OE and UCP4 cells vs KO

	Rescued			UCP4		
Methylation	Hyper	Hypo	All	Hyper	Hypo	All
Full	1130	857	1987	1456	633	2089
Partial	2731	667	3398	1729	990	2719
No	4391	1816	6207	5161	1860	7021

E

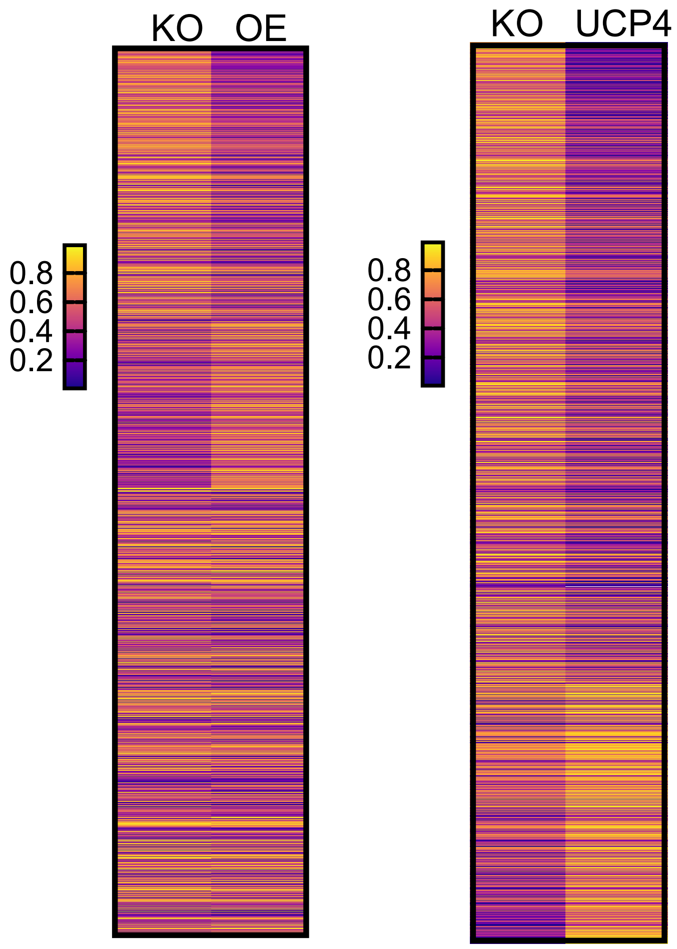

Supplementary Figure 5

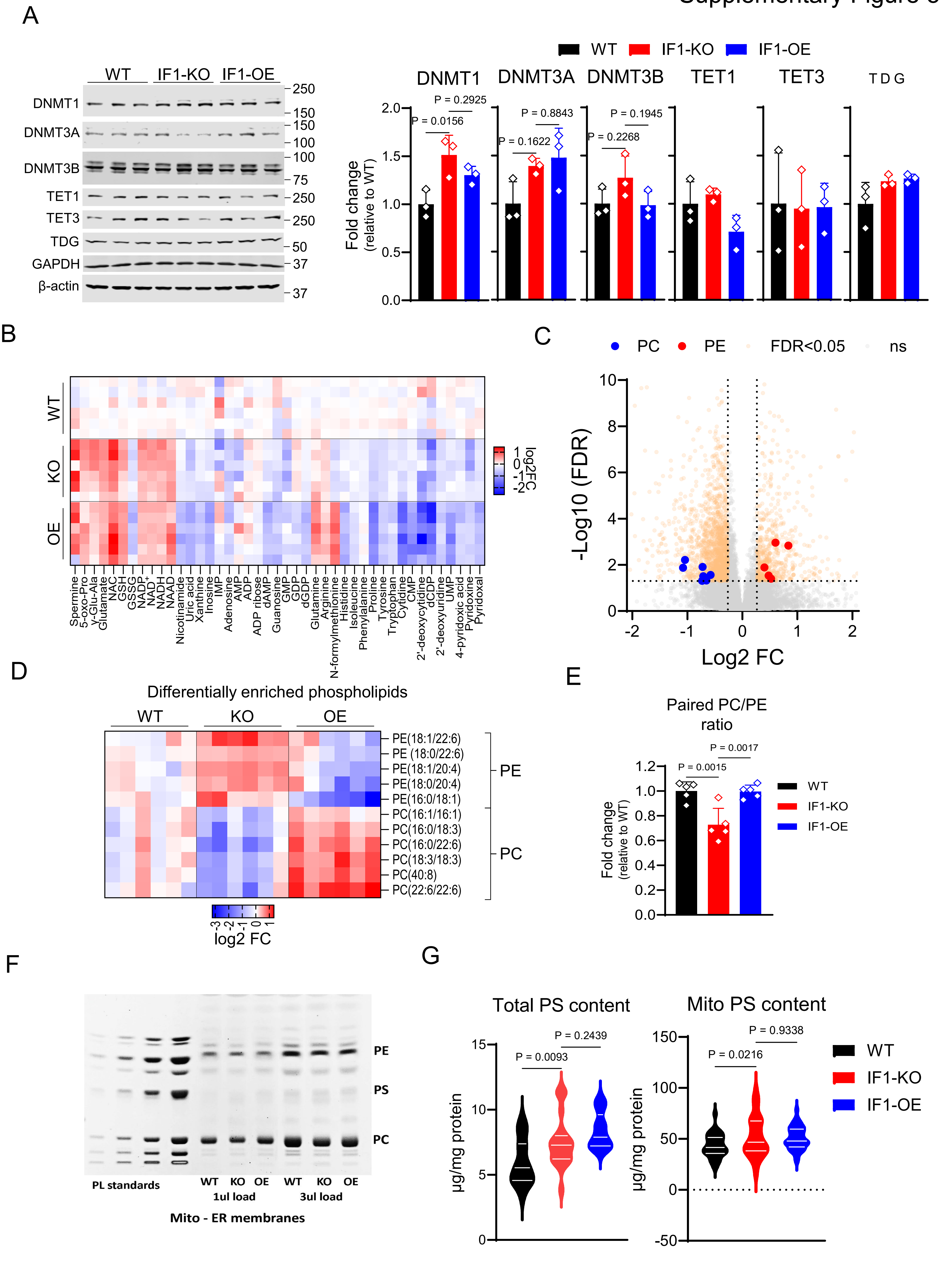

Supplementary Figure 5 - contd

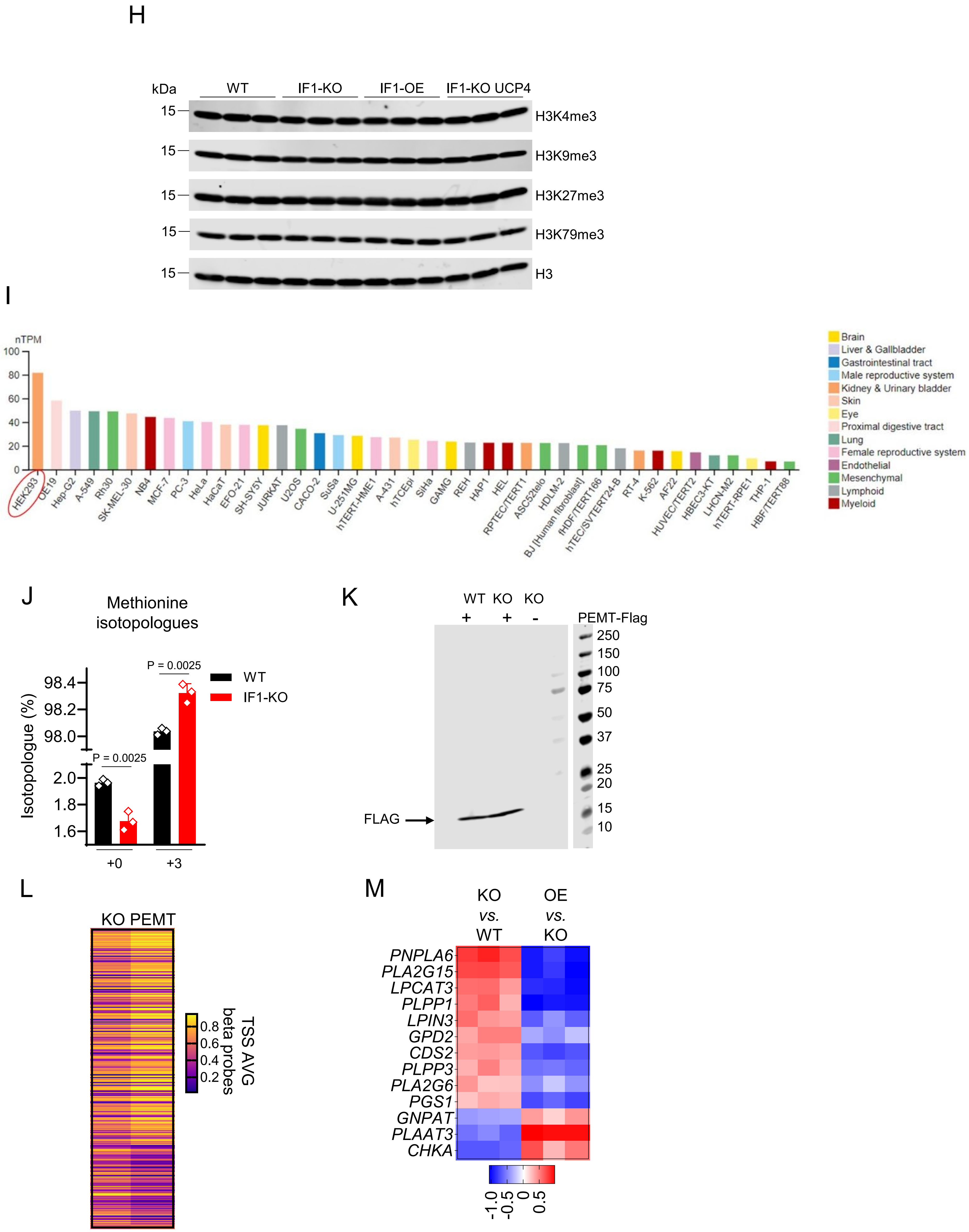

A

Acute (addition with TMRE/MTG or		Chronic (10 days)	
	Simultaneous	Pre-load	
	TMRE	TMRE	Tox21 match
Nigericin	Hyper +++	NA	NA (caused swelling)
Monensin	Hyper ++	NA	Yes
Salinomycin	Hyper +	Hypo --	Yes for acute, no for chronic
Telmisartan	Hyper +	Hyper+	Yes
Annatto	Hyper+	Hyper+	Yes
Nebivolol	Hyper +	NA	Yes for acute, cytotoxic if chronic
Niclosamide	Hypo ---	NA	Yes
Doxorubicin	No difference	NA	No
Bryostatin 1	No difference	NA	No

B

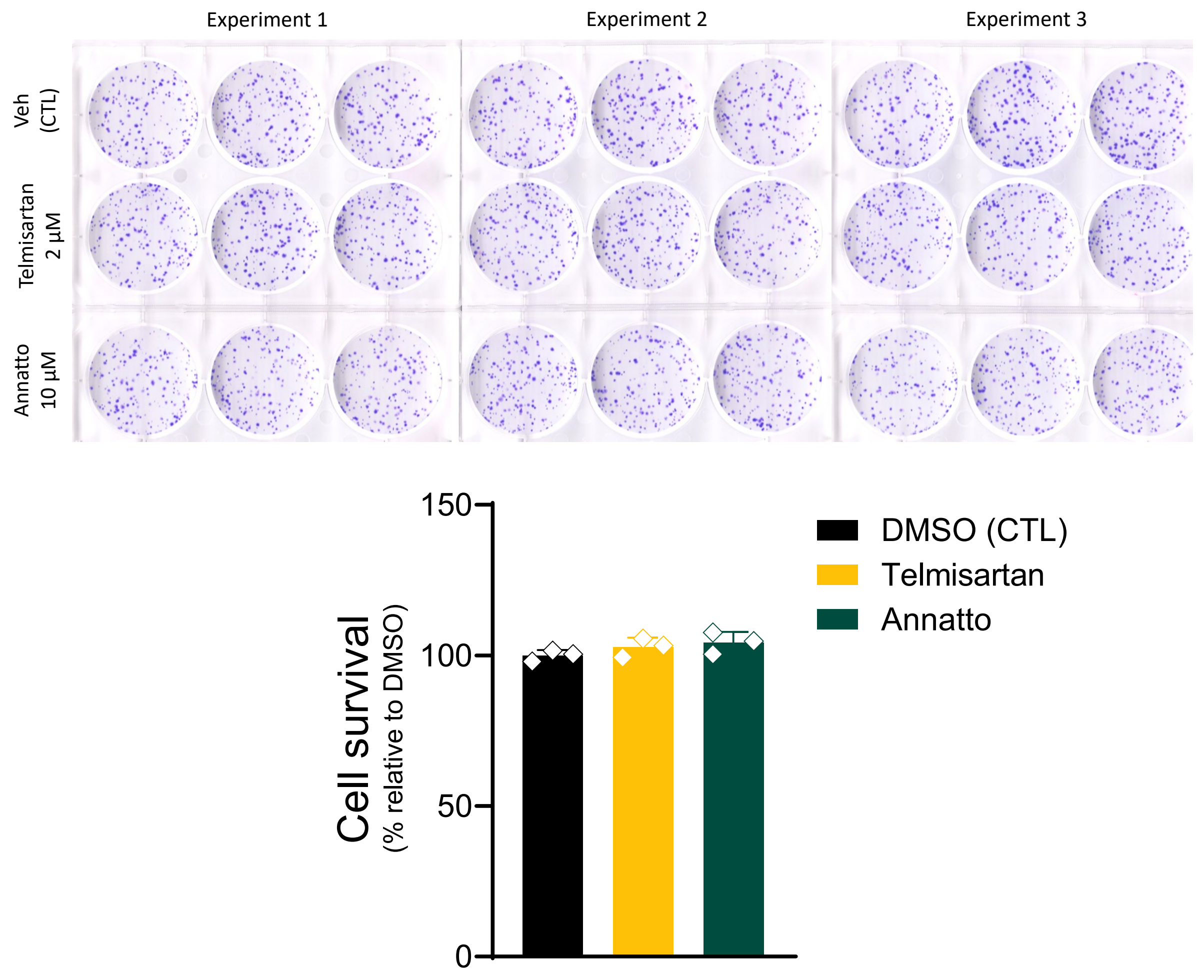

Supplementary Figure 7

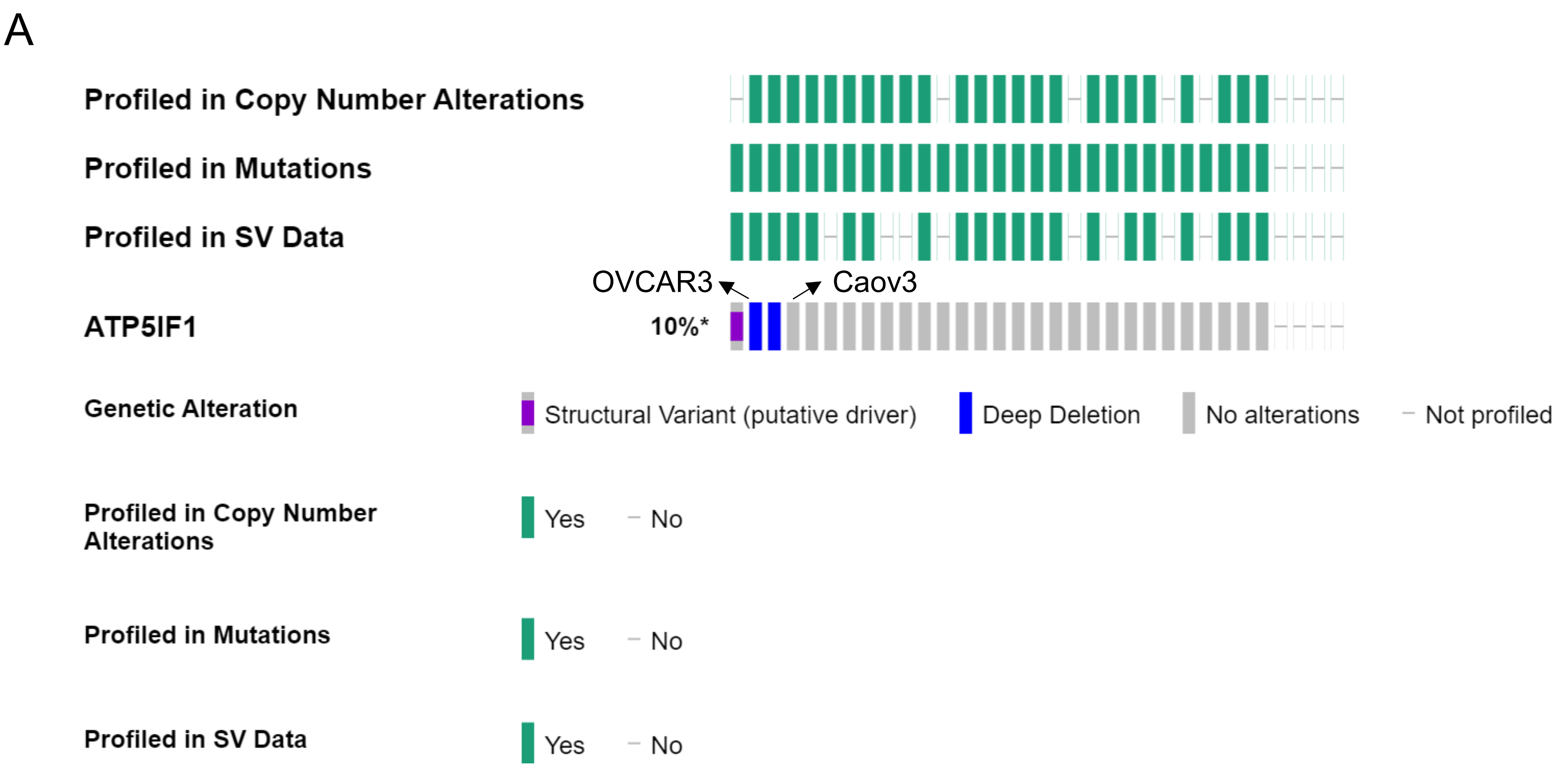

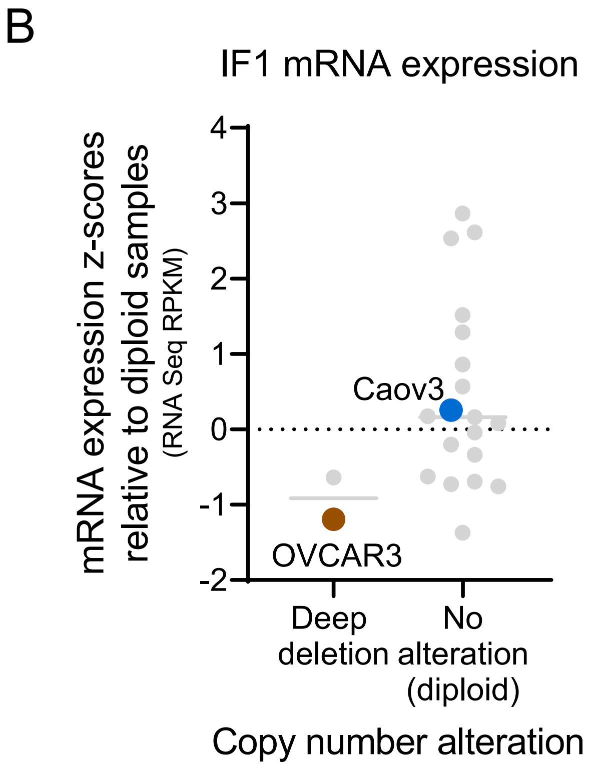

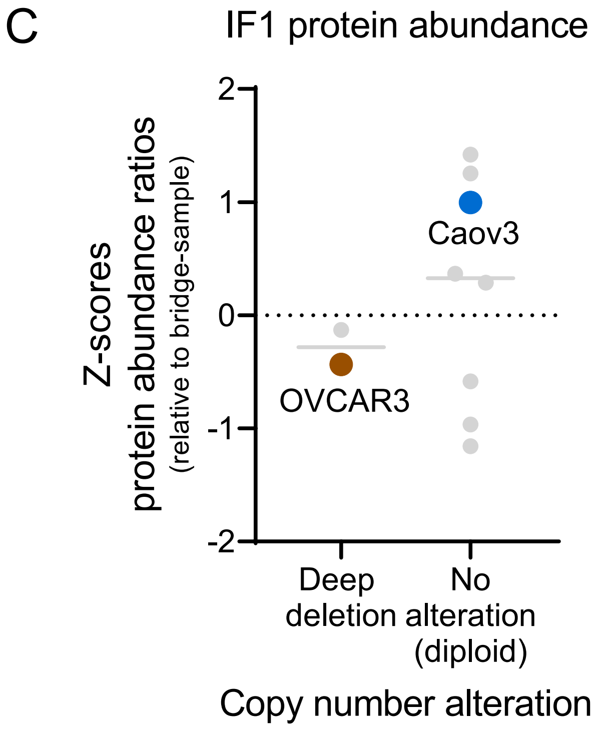

Supplementary Figure 8

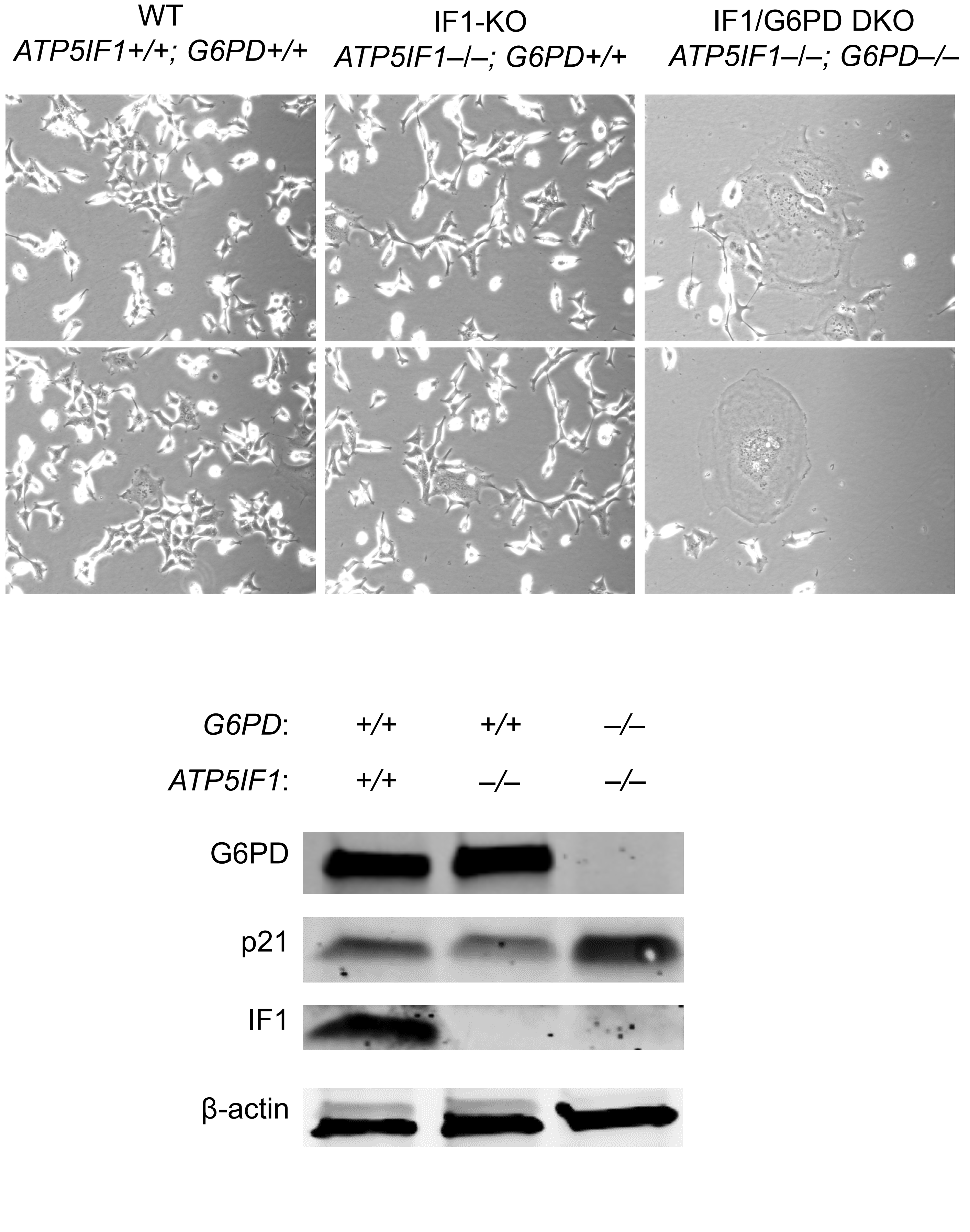

Supplement: Supplementary file 1 — Supplementary Information [file 41467_2025_59427_MOESM1_ESM.pdf]
