# Supplementary material for: Mitochondrial membrane hyperpolarization modulates nuclear DNA methylation and gene expression through phospholipid remodeling
Source: Nat Commun. 2025 Apr 29;16:4029. doi: 10.1038/s41467-025-59427-5 (PMC12041266; doi:10.1038/s41467-025-59427-5)
Supplement: Supplementary file 3 — Description of Additional Supplementary Files [file 41467_2025_59427_MOESM3_ESM.pdf]

Description of additional supplementary files

File name: Supplementary Data 1

Description: RNA-seq data

File name: Supplementary Data 2

Description: DNA methylation data WT vs KO

File name: Supplementary Data 3

Description: DNA methylation data UPC4, OE, PEMT

File name: Supplementary Data 4

Description: TE and H3K9me3 analyses
