# Supplementary material for: Mitochondrial membrane hyperpolarization modulates nuclear DNA methylation and gene expression through phospholipid remodeling
Source: Nat Commun. 2025 Apr 29;16:4029. doi: 10.1038/s41467-025-59427-5 (PMC12041266; doi:10.1038/s41467-025-59427-5)
Supplement: Supplementary file 8 — Reporting Summary [file 41467_2025_59427_MOESM8_ESM.pdf]

nature portfolio

Corresponding author(s): Janine Hertzog Santos

Last updated by author(s): Apr 21, 2025

Reporting Summary

Nature Portfolio wishes to improve the reproducibility of the work that we publish. This form provides structure for consistency and transparency in reporting. For further information on Nature Portfolio policies, see our Editorial Policies and the Editorial Policy Checklist.

Statistics

For all statistical analyses, confirm that the following items are present in the figure legend, table legend, main text, or Methods section.

n/a	Confirmed	
☐	☒	The exact sample size (*n*) for each experimental group/condition, given as a discrete number and unit of measurement
☐	☒	A statement on whether measurements were taken from distinct samples or whether the same sample was measured repeatedly
☐	☒	The statistical test(s) used AND whether they are one- or two-sided<br>*Only common tests should be described solely by name; describe more complex techniques in the Methods section.*
☒	☐	A description of all covariates tested
☐	☒	A description of any assumptions or corrections, such as tests of normality and adjustment for multiple comparisons
☐	☒	A full description of the statistical parameters including central tendency (e.g. means) or other basic estimates (e.g. regression coefficient) AND variation (e.g. standard deviation) or associated estimates of uncertainty (e.g. confidence intervals)
☐	☒	For null hypothesis testing, the test statistic (e.g. *F*, *t*, *r*) with confidence intervals, effect sizes, degrees of freedom and *P* value noted<br>*Give P values as exact values whenever suitable.*
☒	☐	For Bayesian analysis, information on the choice of priors and Markov chain Monte Carlo settings
☒	☐	For hierarchical and complex designs, identification of the appropriate level for tests and full reporting of outcomes
☒	☐	Estimates of effect sizes (e.g. Cohen's *d*, Pearson's *r*), indicating how they were calculated

Our web collection on statistics for biologists contains articles on many of the points above.

Software and code

Policy information about availability of computer code

Data collection	FACSDiVa software (Becton Dickinson) was used to collect all FACS data;
Data analysis	The following commercial programs were used to perform data and statistical analyses:<br>- Microsoft Excel 365<br>- GraphPad Prism 8.0.2<br>- CAMAG automated AMD2 multi-development system was used for HPTLC data analysis;<br>- Compound Discoverer 3.3.0.550 was used for metabolomics analysis;<br>- Methylation analysis was performed with the R Bioconductor package ChAMP v2.22.0

For manuscripts utilizing custom algorithms or software that are central to the research but not yet described in published literature, software must be made available to editors and reviewers. We strongly encourage code deposition in a community repository (e.g. GitHub). See the Nature Portfolio guidelines for submitting code & software for further information.

Data

Policy information about availability of data

All manuscripts must include a data availability statement. This statement should provide the following information, where applicable:

- Accession codes, unique identifiers, or web links for publicly available datasets
- A description of any restrictions on data availability
- For clinical datasets or third party data, please ensure that the statement adheres to our policy

Data supporting the findings of this manuscript are available as supplemental data; Source data, provided as a Source Data file, were included with the submission Genomics data were deposited at the GEO and can be accessed through GSE 295015.

Research involving human participants, their data, or biological material

Policy information about studies with human participants or human data. See also policy information about sex, gender (identity/presentation), and sexual orientation and race, ethnicity and racism.

Reporting on sex and gender	Human subjects were not used in this study
Reporting on race, ethnicity, or other socially relevant groupings	no human data
Population characteristics	no human data
Recruitment	no recruitment conducted
Ethics oversight	not relevant to the current study

Note that full information on the approval of the study protocol must also be provided in the manuscript.

Field-specific reporting

Please select the one below that is the best fit for your research. If you are not sure, read the appropriate sections before making your selection.

☒ Life sciences ☐ Behavioural & social sciences ☐ Ecological, evolutionary & environmental sciences

For a reference copy of the document with all sections, see nature.com/documents/nr-reporting-summary-flat.pdf

Life sciences study design

All studies must disclose on these points even when the disclosure is negative.

Sample size	No a priori calculations were performed. Sample size was chosen based on previous experience with similar measurements.
Data exclusions	No data were excluded from the analyses.
Replication	Experiments in cell lines were performed at least in three independent times, including with independent clones. There were no attempts at replication that failed.
Randomization	Sample allocation was random.
Blinding	Biochemical analyses were not blinded because experiments were performed and analyzed by the same researchers; genomics data were analyzed by either a bioinformatician or Core facility directors that had no prior knowledge about the samples other than their labels.

Reporting for specific materials, systems and methods

We require information from authors about some types of materials, experimental systems and methods used in many studies. Here, indicate whether each material, system or method listed is relevant to your study. If you are not sure if a list item applies to your research, read the appropriate section before selecting a response.

Materials & experimental systems

n/a	Involved in the study	
☐	☒	Antibodies
☐	☒	Eukaryotic cell lines
☒	☐	Palaeontology and archaeology
☒	☐	Animals and other organisms
☒	☐	Clinical data
☒	☐	Dual use research of concern
☒	☐	Plants

Methods

n/a	Involved in the study	
☒	☐	ChIP-seq
☐	☒	Flow cytometry
☒	☐	MRI-based neuroimaging

Antibodies

Antibodies used

ATP5O (Cell Signaling, 92658, 1:1000), ATPIF1 (Millipore, ABC137, 1:400), MICU1 (Sigma, HPA034780, 1:500), MICU2 (Abcam, ab101465 1:500 and Bethyl, A300-BL 19212, 1:1000), MCU (Sigma-Aldrich, AM Ab91189, 1:1000), EMRE (Bethyl, A300-BL 19208,1:1000), β-actin (Cell Signaling, 4967, 1:1000), GAPDH (Sigma-Aldrich, G8795, 1:100), UCP4 (SLC25A27) (Invitrogen, PA5-69265, 1:400), H3K4me3 (ActiveMotif, 39159, 1:1000), H3K9me3 (ActiveMotif, 39161, 1:1000), H3K27me3 (ActiveMotif, 39156, 1:1000), H3K79me3 (Cell Signaling, 4260, 1:1000), H3 (ActiveMotif, 61799, 1:1000), DNMT1 (Cell Signaling, 5032, 1:500), DNMT3A (Cell Signaling, 49768, 1:500), DNMT3B (Cell Signaling, 57868, 1:500), and TET3 (Cell Signaling, 57868, 1:500). Secondary antibodies and dilutions: goat-anti rabbit IRDye 680RD (LI-COR 925-681817, 1:10,000), and goat-anti mouse 680RD (LI-COR 925-68070, 1:15,000).

Validation

All antibodies are routinely used in our lab, negative controls utilized included KO controls and no primary antibodies to identify specificity.

Eukaryotic cell lines

Policy information about cell lines and Sex and Gender in Research

Cell line source(s)

Experiments were performed in HEK293carrying a tetracycline (Tet)-on inducible DN-POLG and the ATPIF1 KO isogenic derivatives were provided by Dr. Navdeep Chandel (Northwestern University), HeLa cells (ATCC cat# : CCL-2); NIH:OVCAR-3, ATCC cat # HTB-161TM) and Caov3 (Caov-3, ATCC cat # HTB-75TM

Authentication

HeLa cells were authenticated by the original supplier (LGC-standards).

Mycoplasma contamination

Cell lines are tested for mycoplasma contamination approximately every six months at the NIEHS Quality Control Core. Cell lines resulted "negative" were used for the study

Commonly misidentified lines
(See ICLAC register)

No commonly misidentified cell lines were used.

Plants

Seed stocks

no seed stocks used

Novel plant genotypes

no plants used

Authentication

not relevant to the study

Flow Cytometry

Plots

Confirm that:

☒ The axis labels state the marker and fluorochrome used (e.g. CD4-FITC).

☒ The axis scales are clearly visible. Include numbers along axes only for bottom left plot of group (a 'group' is an analysis of identical markers).

☐ All plots are contour plots with outliers or pseudocolor plots.

☒ A numerical value for number of cells or percentage (with statistics) is provided.

Methodology

Sample preparation	ΔΨm was accessed using tetramethylrhodamine ethyl ester (TMRE). 500,000 cells were plated in a 6-well plate (56,000 cells/cm2) and incubated at 37 °C in a 5% CO2 humidified incubator for 24 h. Medium was removed and washed once with FBS-free DMEM. Cells were incubated in 2 mL FBS-free DMEM with TMRE 25 nM and MitoTracker® Green (MTG) 50 nM at 37 °C for 15 min. Alternatively, MitoTracker® DeepRed (MTDR) 100 nM was used since HEK293T IF1-OE cells express EGFP. MTG/TMRE-containing medium was removed and gently washed once with complete DMEM. Cells were resuspended incubating cell with TryPLE Express for 2 min at 37 °C and inhibited with complete DMEM.<br>For FACS, approximately 160-240 million HEK293 cells were loaded with MTG 50 nM and TMRE 25 nM as described above. Cells were washed once, counted, and cell density adjusted to 4-5 million cells/mL in complete DMEM with TMRE 10 nM for prolonged time due to sorting. Alternatively, 20-30 million cells from 4 independently growing 150 mm dishes were sorted following the same protocol to access mitochondrial function.
Instrument	BD LSRFortessa and BD Symphony S6 cell sorter (Becton Dickinson Biosciences, San Jose, CA)
Software	FACSDiVa
Cell population abundance	For MMP-based FACS, mitochondrial function was access using MitoStress test in XFe96 Seahorse Extracellular Flux Analyzer.
Gating strategy	For both flow cytometer and FACS experiments, an initial "scatter" gate was set on a forward scatter (FSC-A) versus side scatter (SSC-A) dot plot to isolate the principal population of cells free of debris. Subsequently, cells were consecutively gated on a side scatter height (SSC-H) versus width (SSC-W), then a forward scatter height (FSC-H) versus width (FSC-W) dot plot to isolate single cells. For MMP-based FACS, the center 30% (MFI) of cells stained with MTG (Ex: 488; Em: 537) were gated and examined on a TMRE (Ex: 561/ Em: 585) histogram. The bottom and top 5%, along with the center ~10% of TMRE stained cells, where then collected.

☒ Tick this box to confirm that a figure exemplifying the gating strategy is provided in the Supplementary Information.
